# Evaluation of a large scale implementation of disease management programmes in various Dutch regions: a study protocol

**DOI:** 10.1186/1472-6963-11-6

**Published:** 2011-01-10

**Authors:** Karin MM Lemmens, Maureen PMH Rutten-Van Mölken, Jane M Cramm, Robbert Huijsman, Roland A Bal, Anna P Nieboer

**Affiliations:** 1 Institute of Health Policy and Management, Erasmus University Rotterdam, P.O. Box 1738, 3000 DR Rotterdam, the Netherlands

## Abstract

**Background:**

Disease management programmes (DMPs) have been developed to improve effectiveness and economic efficiency within chronic care delivery by combining patient-related, professional-directed, and organisational interventions. The benefits of DMPs within different settings, patient groups, and versions remain unclear. In this article we propose a protocol to evaluate a range of current DMPs by capturing them in a single conceptual framework, employing comparable structure, process, and outcome measures, and combining qualitative and quantitative research methods.

**Methods:**

To assess DMP effectiveness a practical clinical trial will be conducted. Twenty-two disease management experiments will be studied in various Dutch regions consisting of a variety of collaborations between organisations and/or professionals. Patient cohorts include those with cardiovascular diseases, chronic obstructive pulmonary disease, diabetes, stroke, depression, psychotic diseases, and eating disorders. Our methodological approach combines qualitative and quantitative research methods to enable a comprehensive evaluation of complex programmes. Process indicators will be collected from health care providers' data registries and measured via physician and staff questionnaires. Patient questionnaires include health care experiences, health care utilisation, and quality of life. Qualitative data will be gathered by means of interviews and document analysis for an in depth description of project interventions and the contexts in which DMPs are embedded, and an ethnographic process evaluation in five DMPs. Such a design will provide insight into ongoing DMPs and demonstrate which elements of the intervention are potentially (cost)-effective for which patient populations. It will also enable sound comparison of the results of the different programmes.

**Discussion:**

The study will lead to a better understanding of (1) the mechanisms of disease management, (2) the feasibility, and cost-effectiveness of a disease management approach to improving health care, and (3) the factors that determine success and failure of DMPs. Our study results will be relevant to decision makers and managers who confront the challenge of implementing and integrating DMPs into the health care system. Moreover, it will contribute to the search for methods to evaluate complex healthcare interventions.

## Background

Chronic diseases such as cardiovascular diseases (CVD), diabetes, cancer and respiratory diseases are major causes of death and disability worldwide and their prevalence is expected to rise [1]. They pose a significant health threat and an increasing challenge to health care systems. Despite advances in treatment, research shows that patients often do not receive desirable or necessary care [2].

The causes of chronic diseases are complex and the response needs to be multi-faceted [3]. Disease management programmes (DMPs) have been developed to improve effectiveness and economic efficiency of chronic care delivery [4] by combining patient-related, professional-directed, and organisational interventions [5]. The chronic care model (CCM) clusters six interrelated components of health care systems to transform chronic disease care from acute and reactive to proactive, planned, and population-based [6]. The effectiveness of multifaceted DMPs is the focus of our study.

Introducing complex, multi-component interventions is essentially a process of change [7], but description and explanation of the effects of ongoing DMPs are thus far inadequate. Improvement in terms of care and costs has been documented [8,9], but results vary widely across health care settings, diseases, and target groups. Heterogeneity exists in descriptions of interventions and methodological features such as length of follow-up, outcome measures, and study design [10]. Indeed, the assertion that results are inconsistent is a typical conclusion from an experimental design in the area of disease management. The effectiveness of these programmes is sensitive to an array of influences: leadership, changing environments, details of implementation, organisational history, financial incentives, and more [11,12]. Although traditional (quasi-)experimental methods are important for learning whether improvement interventions change behaviour, they are ineffective in addressing the crucial pragmatic (or "realist") questions about improvement that stem from its complex social nature: what is it about the mechanism of a particular intervention that works, for whom, and under what circumstances [13]? Such questions call for embracing a wider range of scientific methodologies. To be valuable for decision making on local and national levels, evaluation should obtain information on both mechanisms (i.e., how specific social programmes actually produce social changes) and contexts (i.e., local conditions influencing the outcomes) [5]. In response, Glasgow et al. [14] have called for the evaluation of DMPs through practical clinical trials conducted in multiple representative settings, the inclusion of diverse patient groups, the comparison of alternative versions of programmes, and employing multiple measures of relevance to patients, clinicians, and policymakers. Such practical trials should be accompanied by thorough and in depth qualitative analysis of organisational and professional processes in order to explain outcomes.

In the light of the above considerations, the aim of this study is to evaluate an ongoing national network of DMPs by capturing them in a single conceptual framework [5] and using similar structure, process, and outcome measures. The strategy will enable sound comparison of the different programmes. Differences between DMPs, in terms of (combinations of) interventions, organisational factors, context, and degree of implementation are assessed to estimate their independent contribution to programme results. Disease-specific characteristics will be assessed to analyse if they affect DMPs' effectiveness. The study will lead to a better understanding of the mechanisms of disease management (components) and add to the knowledge about the feasibility and cost-effectiveness of a disease-management approach to improve health care. The study will also lead toward identifying factors that determine DMPs' successes or failures. On a meta-level, the study will also improve our understanding of how to evaluate complex interventions like DMPs.

## Methods

### Setting

The study is in the context of a national programme on "disease management of chronic diseases" carried out by ZonMw (Netherlands Organisation for Health Research and Development) and commissioned by the Dutch Ministry of Health. It will focus on the evaluation of the implementation of 22 DMPs to enhance knowledge on disease-management experiments in chronic care, and stimulate implementation of knowledge and insights of successful programmes. The DMPs (see Additional file 1) were selected by ZonMw based on quality and relevancy criteria retrieved from their project proposals, were implemented in various Dutch regions, and comprise a variety of collaborations between organisations and/or professionals (collaborations between general practices and hospitals, primary care practices (including physiotherapists and dieticians), or primary and community settings). The implementation is financially supported by ZonMw and DMPs will receive compensation for participating in the research.

### Patient groups

DMPs target different patient populations, including CVD (9 DMPs), chronic obstructive pulmonary disease (COPD) (5), diabetes (3), heart failure (1), stroke (1), depression (1), psychotic diseases (1) and eating disorders (1). To evaluate the effectiveness of DMPs in real-life settings, we will enrol a broad and representative sample of patients from each DMP. All patients in the DMP will be eligible to participate in the evaluation and no additional inclusion criteria beyond that of the DMPs will be applied [15]. Written informed consent will be obtained from all participants.

### Ethical approval

The study protocol was approved by the ethics committee of the Erasmus University Medical Centre of Rotterdam (September 2009). All personal identifiers will be removed or disguised so the person(s) described are not identifiable and cannot be identified through the details of the story. All eligible patients will receive a brochure with research information and an invitation to participate. The informed consent form makes clear that any patient can terminate participation in the study at any time without consequence to quality of usual care.

### Disease management programmes

**Figure 1:**
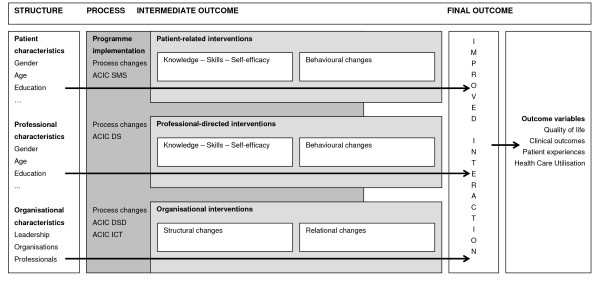
**Relational model**.

Each DMP consists of a combination of patient-related, professional, and organisational interventions (see figure 1). The exact programme components for each region may vary. The core of a DMP is described below; for detailed programme information, see the Additional file 1.

#### Patient-related interventions

Self-care is critical to optimal management of chronic diseases. Patients empowered and effective in self-management are better prepared to positively influence disease control and health outcomes [16]. Hence, all 22 DMPs include such interventions. Examples of self-management within the DMPs are patient education on lifestyle, motivational interviewing, regulatory skills, and proactive coping.

#### Professional-directed interventions

Care standards, guidelines, and protocols are essential parts of DMPs. They must be integrated through timely reminders, feedback, and other methods that increase their visibility at the time that clinical decisions are made [17]. All DMPs are built on (multidisciplinary) guidelines. Those directed at CVD will also implement the CCM; a care standard for diabetes is available. The implementation strategies for professional interventions may, however, vary. All DMPs provide training for their professionals. Implementation of the guideline in 19 DMPs was supported by ICT tools such as integrated information systems.

#### Organisational interventions

Effective DMPs require organisational changes. This often includes organising new collaborations of care providers, allocating tasks differently, transferring information and scheduling appointments more effectively, case management, using new types of health professionals [5,18], redefining professionals' roles and redistributing their tasks, planned interaction between professionals, and regular follow-up meetings by the care team. Many forms of organisational changes will be applied in the 22 regions.

### Research methods

The study is a practical clinical trial because it addresses questions about the risks, benefits, and costs of DMPs as they would occur in routine clinical practice [15]. Moreover, it combines qualitative and quantitative research methods [19]. The application of a previously developed evaluation model will allow us to determine the relationships between interventions and outcomes [5]. Such a design will yield empirical evidence on the effects of the DMPs as well as reveal effective aspects of the programme and care process changes. Furthermore, it will give insight in working DMPs and demonstrate which elements of the intervention are potentially (cost-) effective. Hence, it will be possible to uncover opportunities and threats to the implementation of DMPs.

**Figure 2:**
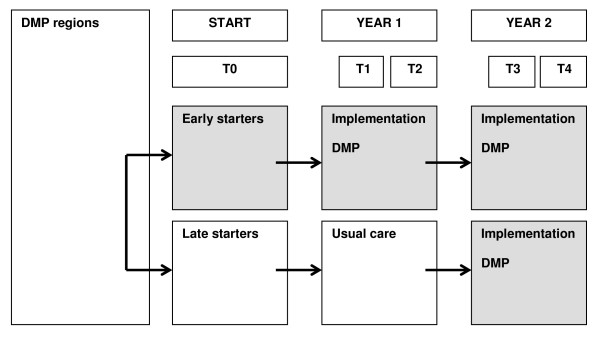
**Study design**.

The main comparison will be between DMPs, but most have a pre-post test design within them. Seven DMPs have a control group of patients for whom the intervention will be postponed by a period of 1 year (late starters). General practices (clusters) will be assigned to an early starter (intervention) group or late starter (control) group (see figure 2). Two DMPs concerned with eating disorders and stroke will assign patients at random to the comprehensive DMP or usual care.

### Process evaluation

The effectiveness of a DMP is highly dependent on successful implementation, the understanding of which requires insight into the "black box," or sequence of events through which subjects are affected by the interventions [5]. Glasgow et al. [20] proposed measurement of contextual factors, intervention implementation, and behavioural change at both patient and staff levels (see figure 1). The research question is: "What interventions are performed within the context of the 'disease management in chronic diseases' programme?"

Process evaluation requires measurement instruments that are sensitive to specific interventions and connected to expected changes in outcome data. We will use multidisciplinary guidelines, care standards, and project protocols to define process indicators and monitor programme implementation (see table 1). Data will be collected from health care providers' data registries. For example, we would compare whether actual follow-up appointments equalled those recommended by the guidelines.

**Table 1:** Effect evaluation: outcome variables and instruments

Outcomes variables	Instruments	Items/value
**Quality of life**		
SF-36	Validated questionnaire	36 items
EQ-5D	Validated questionnaire	5 items
VAS	Validated questionnaire	1 item
HADS	Validated questionnaire	14 items
**Patient experiences**		
PACIC	Validated questionnaire	20 items
**Health care utilisation**		
Health care utilisation	Questionnaire	18 items
Medication utilisation	Questionnaire	5 items
Health and Labour (SF-HLQ)	Validated questionnaire	7 items
**Clinical outcomes**		
HbA1c (diabetes)	Medical registries	%
Glucose (diabetes/CVD)	Medical registries	mmol/l
Blood pressure (diabetes/CVD/heart failure)	Medical registries	mmHg
Lipids (diabetes/CVD)	Medical registries	
LDL		mmol/l
Total cholesterol		mmol/l
FEV_1 _(COPD)	Medical registries	%
FEV_1_/FVC (COPD)	Medical registries	
Exacerbations (COPD)	Medical registries	
BDI (depression)	Medical registries	

**Intermediate variables**		

Smoking/Smoking behaviour	Medical registries/Validated questionnaire	smoking status 4 items
Physical Activity/SQUASH	Medical registries/Validated questionnaire	5 items
Diet	Medical registries	
SMA-S	Validated questionnaire	15 items

**Process variables**		

ACIC	Validated questionnaire	34 items
PSAT	Validated questionnaire	84 items
Relational Coordination Survey	Validated questionnaire	8 items
Motivation of professionals	Validated questionnaire	32 items
Process measures (e.g. % patients with care plan)	Medical registries	%

Notes: SF-36 = Short Form 36; EQ-5D = EuroQol; VAS = Visual Analogue Scale; HADS = Hospital Anxiety and Depression Scale; PACIC = Patient Assessment of Chronic Illness Care; CVD = cardiovascular disease; COPD = chronic obstructive pulmonary disease; SMA-S = Self Management Ability Scale; ACIC = Assessment of Chronic Illness Care; PSAT = Partnership Self-Assessment Tool; SF-HLQ = Short Form-Health and Labour Questionnaire; SQUASH = Short Questionnaire to Assess Health-Enhancing Physical Activity.

Process implementation and contextual information will also be measured by physician and staff questionnaires. The extent to which the care delivered in each project is consistent with the CCM will be measured with the Assessment of Chronic Illness Care questionnaire (ACIC) [21]. In projects targeting different diseases, the instrument will enable comparisons. The questionnaires will also indicate how closely organisational structures and processes reflect the components of disease management [12], coordination mechanisms between organisations and professionals, measures of leadership and participation, and so on.

Qualitative data will be gathered via interviews with all 22 project leaders and via document analysis for in depth descriptions of project interventions and the contexts in which DMPs are embedded. An ethnographic design [22] will be used in five DMPs, since ethnographic process evaluations of the implementation of interventions and their adaptation in practice are necessary to assess the validity and reliability of any intervention effects [23]. Baseline descriptions of the DMPs were used to select five cases (see Additional file 1) which reflect variety in regions, patient groups, and interventions. The essence of thick descriptions is to provide a layered and in depth understanding in order to draw conclusions and uncover the intentions of the actual interventions. First, we will seek thick descriptions to (1) understand how disease management is enacted in practice [24], (2) gain better understanding of cooperation between project partners, and (3) reveal possible effects of contextual factors such as finance and culture. The qualitative analysis will also focus on how measurements configure the relations between organisations, professionals, and professionals/patients because research has shown the importance of measurements in quality improvement [25]. Second, within each of the five projects, ten to fifteen interviews, depending on size and scope of the project, will be held with project managers, participating health professionals, and patients. We will also analyse documents to study regional histories of integrated care. Third, we will observe project meetings to analyse interactions between project partners. Qualitative data collection of the five cases will be repeated several times to analyse project dynamics and changes over time; after a baseline description at the onset of the projects, project managers will be interviewed every six months. A more elaborate round of ethnographic analysis will be done after implementation of the DMPs.

### Health outcomes

The aim of the evaluation will be to determine whether the DMP has achieved the intended effects on processes and outcome indicators in the intermediate (e.g., lifestyle and behaviour) and final (clinical parameters, quality of life (QoL), health care utilisation, and patient experiences) stages. The research question is, "What are the effects of DMP on outcomes at the patient, professional, and organisational levels?"

Tunis et al. [15] have called for increased reporting on outcomes relevant to decision makers, including QoL, symptom severity, satisfaction, and cost. Table 1 gives a detailed overview of the outcome measures and instruments. A core set of outcomes will be measured using a self-administered questionnaire including QoL, health care utilisation, and patient experiences. We will also measure disease-specific clinical outcomes (table 1) by inspecting data from health care providers' data registries, preferably electronic ones (e.g., EPD or GP information systems (HIS)). We will collaborate closely with DMP project leaders and local data managers in collecting data.

### Economic evaluation

Cost-effectiveness of DMPs is an important component of evaluation in lieu of tight health care budgets and high costs of chronic care. The research question is "What are the total costs, including those of development, implementation, and health care utilisation, associated with the interventions; how are they financed and reimbursed; and how do they relate to the effects of the interventions?" Each DMP will be subjected to a full economic evaluation conducted from societal and health care perspective using the same methodological framework across DMPs.

Costs associated with the development and implementation of interventions are collected using a standardised (Excel) format that incorporates costs of health care professionals, support staff and management, as well as costs of ICT, training, information and communication, material, travel, overhead, and so on. These data are provided by each DMP project leader and finalised by the HTA-researcher during site visits to each project. Patient health care utilisation will be collected through an adapted version of a standardised questionnaire that concerns contact with care providers, hospital admissions, distance travelled, medication use, and work absenteeism. The last component enables us to estimate costs of productivity losses due to illness. In a limited number of DMPs (see Additional file 1: programmes no. 6, 7, 14, 21, and 22) with severely ill patients, we will measure the costs of informal care. The sum of the development and implementation costs plus the costs of health care utilisation are the total costs of a DMP.

### Timing of measurements

**Figure 3:**
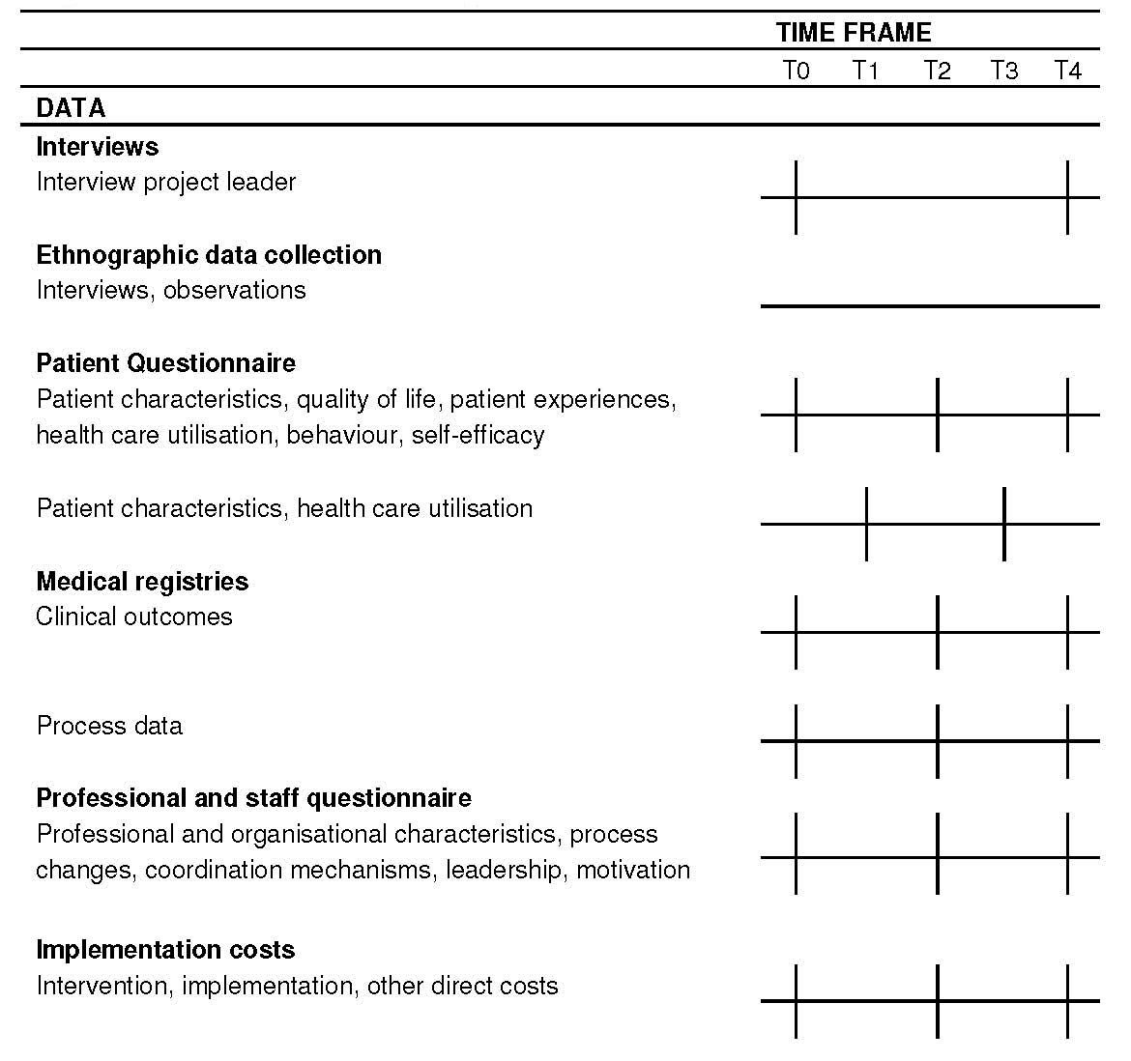
**Timeframe of the study**.

Information will be collected at five moments over a period of two years: intervention baseline (T0) and each 6 months thereafter (T1-T4) until two years after the start of the DMP (figure 3). At T0, the research team will collect data from patient records such as lung function, blood pressure, and lipid values for clinical outcome and process measurement. A sample of patients participating in the DMP will receive the first questionnaire from their general practitioners (GPs) to assess patient history, QoL, health care utilisation and patient behaviour. At T1 and T3, patients will receive a short questionnaire to assess health care utilisation only. At T2 and T4 (end), the team will once more collect data from patient records and GPs will again issue patients the questionnaire. Project leaders will provide information on the costs of development and implementation of the DMP at T0, T2, and T4. At the same time points the professional and staff questionnaires will be administered. At T0 and T4 qualitative interviews will be held with all project leaders. Ethnographic data will be collected throughout the implementation process.

### Data analysis

We will explore the effects of DMPs by monitoring their implementation differences. Descriptive statistical analysis will be performed on patient demographics and other study variables, such as combinations of interventions or programme implementation. In order to evaluate the costs and effects of the disease management interventions on the primary outcomes at the patient, professional and organisational levels, we will compare changes in outcome measures at T2, T3, and T4 between DMPs and within programmes between intervention and controls. Primary outcomes in terms of functional status and QoL will be estimated after correction for potential confounders and differences in starting values between the treatment groups or projects. Changes in generic health outcome measures (SF-36, EQ-5D), number of disease episodes (flare-ups), and patient experiences will be comparable across projects with recognition to the fact that the episodes' nature and impact varies across diseases. The primary clinical outcomes (e.g., lung function, cholesterol level) as extracted from medical records are to some extent made comparable across projects by expressing them in number of patients with a 'minimal clinically important' improvement.

All data will be analysed according to intention-to-treat principles in which data from all subjects are used regardless of the subjects' adherence to protocol. To account for missing costs and health outcomes due to incomplete projects and the uncertainty so introduced, we will perform sensitivity analyses using various imputation techniques.

The relation between a DMP's total costs and the estimated health outcome changes is expressed in cost-effectiveness ratios. Examples are costs (1) per QALY, (2) per additional patient with a minimal clinically relevant improvement in QoL, (3) per additional patient with a clinically relevant improvement in the clinical outcome measure, and (4) per disease episode prevented. Uncertainty around cost-effectiveness will be dealt with by bootstrapping costs and health outcomes, plotting them on cost-effectiveness planes, and drawing cost-effectiveness acceptability curves.

A comprehensive investigation at the individual and organisational levels requires multi-level analyses, incorporating variables at different levels of aggregation to differentiate between compositional and contextual effects. Analysis of the outcomes at T1, T2, T3, and T4 will take the dependency of outcomes within patients into account. Multi-level models will be used to distinguish between project- and patient-level effects [26]. The hierarchical structure of the data gives us the opportunity to test the effects of the interventions with respect to structural and process characteristics. Success and failure factors such as the culture of the participating organisations and relational coordination (e.g., communication patterns) are likely to relate to the effectiveness of specific interventions. Multi-level analyses will enable us to test such contextual effects across DMPs.

We will analyse the study's qualitative data - interview transcripts, project documents, and non-participant observation notes - with Atlas.ti. We will use open coding to be alert to issues emerging from the data and if necessary collect additional data to follow themes. Coding will be done by two researchers to increase reliability.

### Integration of findings

Methodologically, the assessment of a DMP is the evaluation of a complex mixture of interventions at the patient, professional and organisational levels. Therefore, qualitative and quantitative methods are mixed throughout all phases of the project from the design stage through data collection and interpretation. This enables understandings of (1) the mechanisms through which DMPs produce change, (2) the contextual conditions necessary to trigger such mechanisms, and (3) the effects of DMPs with respect to context and triggered mechanisms.

Intermediate results of the qualitative, quantitative, and economic analyses will be continually fed back within the research group to improve the 'mixed' character of the study, and enable recognition of emerging themes across research methods and a more fine-grained analysis of data. This is especially relevant in the qualitative component of the project. The quantitative data will be revealed to the qualitative researcher to allow him or her to tailor the interviews and observations accordingly. Although each researcher has responsibility for a particular part of the study, regular team interaction will ensure optimal integration of the results of the different study parts.

## Discussion

The introduction of complex, multi-component interventions is sensitive to an array of influences such as details of implementation and context [11,12] and as such calls for embracing a wide range of scientific methodologies. Although traditional (quasi-)experimental methods are important to identifying whether improvement interventions have changed care outcomes, they reveal little about the underlying mechanisms of effectiveness. And, although descriptive studies provide appropriate understanding of mechanisms and context of change, they lack rigor in terms of understanding effectiveness of the intervention. This article thus presents a methodological approach that combines qualitative and quantitative (mixed) research methods, enabling a thorough and comprehensive evaluation of complex programmes.

While improvement of quality of care and cost-effectiveness has been documented in the Netherlands [27-29], the results vary widely across health care settings, diseases, and target groups. In addition, systematic reviews pooling evidence from different chronic diseases [8,9,30] or a single chronic condition (e.g., heart failure, depression, diabetes, COPD [31-33], [10]) suggest that DMPs are, to some extent, effective. Indeed, the assertion that DMPs do not work or that results are inconsistent and more research is needed is typical of ill-fitting research methods. Interpretation is hampered by differences in the external context of care providers, cultural aspects, implementation problems, and availability of resources [7]. The strength of this study's evaluation plan is its diversity of tools and perspectives. Moreover, the application of a theoretical model improves the design and evaluation of DMPs [5]. Process, intermediate, and final outcome indicators were selected on theoretical grounds. A priori, we expect to find greater changes in the process and intermediate outcomes because they are direct DMP targets. Given the current knowledge on the link between these outcomes and the health outcomes, it is likely that the latter will improve too. It may, however, take longer than two years of evaluation before the full effects become visible. Extrapolating results to longer time periods using decision analytic cost-effectiveness models is an option, provided there is sufficient information on the association between intermediate and final outcomes.

Some study limitations can be described in advance. First, given that the nature of DMPs is 'practical' and the level of control is likely to be lower than randomised controlled trials, we anticipate a higher proportion of missing observations. But the fact that the DMPs are financially supported to participate and provide data may mitigate missing values in data collection. Second, this study will evaluate 22 DMPs, which will generate variation between programmes. General measures given to each site will yield a solid evidence base and, in combination with other data sources such as qualitative descriptions, will lead to knowledge of DMPs' effectiveness. Moreover, variation can be seen as strength, and can be used as an important source of information. Data collection from multiple settings, diverse patient groups, and alternative programme versions, and employment of multiple measures will result in rich data relevant to patients, clinicians, and policymakers [7].

In our study a wide range of scientific methodologies is embraced to evaluate DMPs and obtain information on both mechanisms and contexts that will be valuable for decision making on local and national levels. Therefore, this study will lead to a better understanding of the mechanisms of DMPs and add to the knowledge on the feasibility and cost-effectiveness of DMPs in improving health care. Finally, the study will better highlight the factors that determine the success and failure of DMPs.

## Competing interests

The authors declare that they have no competing interests.

## Authors' contributions

KL, MR, RH, RB and AN participated in the study design. KL and AN were involved in all aspects of the study. JC described the 22 participating DMPs. All authors contributed to the manuscript and have read and approved its final version.

## Pre-publication history

The pre-publication history for this paper can be accessed here:

http://www.biomedcentral.com/1472-6963/11/6/prepub

## Supplementary Material

Additional file 1 **Appendix**. 22 disease management programmes. Click here for file
