# Supplementary material for: Evaluation of a large scale implementation of disease management programmes in various Dutch regions: a study protocol
Source: BMC Health Serv Res. 2011 Jan 10;11:6. doi: 10.1186/1472-6963-11-6 (PMC3025828; doi:10.1186/1472-6963-11-6)
Supplement: Additional file 1 — Appendix. 22 disease management programmes. [file 1472-6963-11-6-S1.DOCX]

| **DMP** | **Disease** | **Region** | **Patient-directed interventions** | **Professionally-directed interventions** | **Organisational interventions** |
| --- | --- | --- | --- | --- | --- |
| 1 | CVD | Amsterdam | -Use of EPD to enhance self-management | -Implementation of care standard for cardiovascular risk management  -Uniform treatment protocols in primary and hospital care  -Increase knowledge and independence of practice nurses  -Benchmarking and auditing of patient satisfaction | -Delegation of care from specialist to nurse practitioner or primary care practice nurse  -Redistribution of hospital to primary care  -Regular follow-up  -Intensification of cooperation with physiotherapists in using existing exercise programmes  -Regional transmural EPD with a patient portal containing information on projects and referral options |
| 2 | CVD | Achterveld | -Training of patient groups in active participation and self-management (healthy diet, exercise, stress management) | -Educational meetings on the DMP  -Education and training in patient stimulation and support to enhance their active involvement  -Setting quality parameters for auditing and feedback | -Delegation of care from GP to practice nurse, from heart specialist to GP  -Regular follow-up  -Integrated information system  -Implementation of regional trans-mural EPD in 3 years |
| 3 | CVD | Amsterdam | -Informational meetings (also in Turkish and Moroccan)  -Community-based lifestyle interventions  -Individual care plans  -Support of self-management with internet, email, text messages, or incentives  -Lifestyle and exercise programmes | -Basing GP primary care protocols on the care standard cardiovascular risk management and NHG standards  -Education and training for lifestyle programmes  -Training cycle for GPs  -Education of practice nurses in CVD care  -Use of the ICT system for benchmarking | -Use of the patient platform to implement self-management programmes  -Communication with local immigrant organisations to identify and mitigate potential barriers to immigrants’ involvement in DMPs  -Redistribution of hospital to primary care  -Delegation of care from specialist to GP or practice nurse  -Regular follow-up  -Transmural care chain  -Cooperative agreements between primary and hospital care  -Special policies for immigrants  -Investigation of (im)possibilities for chain-integrated information system with a patient portal |
| 4 | CVD | Eindhoven | -Patient education  -Motivational training  -Personal coaching  -Facilitation of self-monitoring and self-management  -Customised programmes to quit smoking, exercise, maintain healthy diet and develop coping skills | -DMP education  -Training in motivational training  -Use of validated performance and process indicators as quality parameters for auditing and feedback  -Individual monitoring of patients and evaluating quality of care at group level  -Provision of feedback and suggestions for improvement by the care registration team | -GP as the central care provider in close collaboration with the practice nurse  -Emphasis of care providers’ coaching role  -GP responsibility for primary care patients and ambulatory patients in hospital care  -Specialist responsibility for clinical patients  -Exercise programmes provided by a consultant at regular sports facilities  -Regular follow-up  -Cooperation with diagnostic centres and hospital care specialists  -Cooperative agreements between primary and hospital care  -Early detection of high-risk patients  -Uniform systematic registration by all professionals  -Support of ICT-registration system for monitoring and feedback |
| 5 | CVD | Maarssen | -Lifestyle advisors and plans (exercise, diet, quit smoking)  -Motivational interviewing  -Exercise programmes  -Quit smoking consultation hours  -Development of individual patient care plans based on their risk profiles | -Cardiovascular programme based on the care standard for cardiovascular risk management  -Setting quality parameters for auditing and feedback  -Benchmarking  -Regular intervention and goals evaluation by central care director | -Mapping of patients’ wishes and active involvement of patients or patient groups in the CVD programme  -Annual patient satisfaction inquiry  -Central care director (nurse practitioner) responsible for content of the care plan, delegation of lifestyle interventions to the lifestyle advisor, regular meetings with the pharmacist on patients’ medication use, and proactive contact with other involved professionals  -Collaboration of lifestyle advisor and patient on coaching and lifestyle plans  -CVD practice nurse consultation hours 4 times a week. Patient inflow through GP and active involvement with at-risk patients  -Shared decision making and actively reminding patients of their decisions and treatment plans  -Regular follow-up  -Expansion of chain care to hospital care  -Trans-mural protocol  -Chain-integrated information system with a patient portal |
| 6 | Heart failure | Alkmaar | -Education of patients to enhance self-management skills | -Implementation of CBO trans-mural guidelines  -Implementation of NHG standard in all primary care settings  -Education of professionals involved in the DMP  -Setting quality parameters for auditing and feedback | -Involvement of patient associations  -Delegation of care from GP to practice nurse and heart specialist to GP  -Patient follow-up to improve continuity of care  -Design of a trans-mural care standard or protocol to improve cooperation and provide clear referral agreements between all professionals involved in the DMP  -Communication platform for professionals to discuss patients  -Chain-integrated information system |
| 7 | Stroke | Amsterdam | -Informational meetings  -Lifestyle programmes through motivational interviewing  -Customized exercise programmes | -Implementation of care standard for cardiovascular risk management  -Checklist for risk factors  -Measurement of treatment adherence  -Assessment of patient satisfaction for quality improvement | -Delegation of care from specialist to primary care practice nurse supervised by GP  -Assessment of patient satisfaction for quality improvement  -Lifestyle programmes provided by physical therapist and stroke nurse  -Enhanced secondary prevention led by GP or stroke nurse at the nursing home and monitored by the neurologist  -Regular follow-up  -Multidisciplinary approach in hospital care |
| 8 | CVD | Arnhem | -Joint medical consultations for primary care and hospital care patients (n = 10), a spouse/family member, and a physician | -Improved implementation of guidelines for cardiovascular care in primary and hospital settings  -Implementation of care standard for cardiovascular risk management to improve implementation of CBO and NHG protocols in primary and hospital care settings  -Education for GPs  -Cooperative learning among nurses at the outpatient clinic | -Knowledge exchange  -One-stop outpatient clinic  -Joint consultation hours  -Regular follow-up in primary care  -Cooperation between primary and hospital care  -Uniform treatment plan in primary and hospital care  -Early recognition of high-risk patients at outpatient clinics and general practices  -Transmural EPD vascular risk management with a patient portal  -Development of trans-mural DBC |
| 9 | CVD | Nijmegen | -Patient education on cardiovascular risk management  -Exercise programmes  -Quit smoking programme run by a quit-smoking consultant  -Healthy diet plans  -Health counselling | -Education of a Turkish-speaking practice nurse  -Implementation of a special immigrant protocol  -Auditing and feedback to improve the quality of patient satisfaction and outcome indicators | -Delegation of care from GP to nurse  -Nurse-managed consultation  -Provision of customized, demand-driven care  -Semi-weekly meetings between GP and practice nurse  -Ability of practice nurse to contact professionals from other disciplines (e.g., dietician)  -Regular follow-up  -Special immigrant policy  -New structured and formalized roles and cooperation between nurses, GPs, and other professionals involved in the DMP  -Development of a safe integrated ICT system |
| 10 | CVD | Nijmegen* | -Patient-driven choice programme: patient can choose a central care provider, the risk factor(s) he/she wants to tackle, the intervention(s), personal goals, and use of web-based support  -Individual care plans with personal goals  -Self-management support with vulnerable groups  -Motivational interviewing  -Cognitive behavioural therapy | -Implementation of care standard for cardiovascular risk management  -Education of professionals  -Auditing and feedback sessions to improve quality of patient satisfaction and outcomes  -Email alerts for professionals that identify unmotivated patients | -Internist and vascular nurse as central care providers  -Regular follow-up  -Contact with unmotivated patients every 3 months  -Development of trans-mural collaborative care structure  -Enhanced interaction and cooperation among professionals involved in the DMP on referrals and treatment plans  -Contact with patients of low socioeconomic status and different cultural backgrounds  -Online patient files accessible to all professionals and the patient  -Registration of risk profiles in the ICT system |
| 11 | CVD | Huizen | -Education of patients to enhance self-management skills  -Motivational interviewing  -Individual care plans  -Exercise programmes  -Quit smoking counselling  -Healthy diet counselling  -Web-based support programmes to enhance self-management (access patient file, information, e-consultation) | -Implementation of care standard for cardiovascular risk management  -Education of professionals on informing patients and motivational interviewing  -Education of physician’s assistants on cardiovascular risk factors  -Auditing and feedback on performance indicators and benchmarking | -Consultation with several patient groups  -Patient satisfaction research  -Delegation of care from GP to practice nurse  -Practice nurse as central care provider  -Identification of patient groups based on risk profiles  -Pharmacist monitoring of medication  -Physical therapist-run exercise programmes  -Healthy diet counselling from practice nurse in collaboration with dietician  -Development of multidisciplinary treatment programme for obesity  -Registration of risk profiles  -Structural knowledge exchange  -Regular follow-up  -Cardiovascular counselling hours  -Cooperation between primary and hospital care  -Development of a multidisciplinary programme for obesity  -Electronic self-management tool  -Professional information exchange in the ICT system |
| 12 | COPD | Tilburg* | -Mirror interviews | -Professionals work according to regional COPD guidelines  -Quarterly online distribution of a newsletter and DMP information  -Semi-annual casuistry meetings of lung specialists, GPs, and practice nurses on spirometry and correct interpretation of results  -Regular general practice audits of lung specialists  -Document review of all lung function measures performed at the general practices by lung specialist at the hospital  -Individual and regional feedback from the ICT system | -Regional chain-integrated information system |
| 13 | COPD | Arnhem | -Individual care plans  -Customised exercise programmes  -Customised COPD education  -Quit smoking programmes  -Monitoring self-management during every consultation | -Implementation of CBO and NHG standards  -Ability of GPs to adapt protocols and agreements to their own practices  -Education of involved professionals in COPD and spirometry  -Education of practice nurse in motivational interviewing of COPD patients  -Auditing and feedback on quality of life and  process and outcome measures | -Assignment of COPD physicians and lung nurses as consultants  -Distribution of hospital to primary care  -Chain-integrated information system  -Implementation of (care standard) protocols in all information systems  -Design of a trans-mural care standard or protocol to improve cooperation and provide clear referral agreements between all professionals involved in the DMP |
| 14 | COPD | Monnickendam | -Individual care plans  -Quit-smoking programmes  -Exercise programmes (optional)  -Patient groups to increase disease-related knowledge and self-management skills  -Treatment of psychological problems that inhibit coping skills | -Auditing and feedback on lung function, muscles, exacerbation parameters and quality of life | -Collaboration of practice nurse and COPD patient on care plan  -Regional trans-mural care agreements  -Active follow-up of asthma patients for early detection of COPD  -Development of a care plan for COPD GOLD 3 and 4 patients: policy addressing exacerbations, adherence, exercise programmes, monitoring, and changes in quality of life  -Setting a regional example to facilitate regional continuation of the DMP |
| 15 | COPD  Diabetes  CVD | Boxmeer* | -Individual care plans  -Education of patients through an informational booklet  -General lifestyle programmes  -E-health programmes to improve preventive behaviour, healthy lifestyle and self-management skills  -Group consultations | -Educational meetings on improving prevention and enhancing healthy lifestyles  -Systematic measurement of patient-level outcomes and benchmarking | -Integration of care programmes for diabetes, COPD, and CVD  -Regional network for improved prevention and healthy lifestyles  -Participation in development of centre for prevention and health  -Regional ICT system to support self-management programmes  -Attempt to implement several chain DBCs |
| 16 | COPD | Almere | -Education of patients through an informational booklet, online information, information about patient associations, websites, and meetings  -Four informational meetings providing general information about COPD, multidisciplinary treatment and self-management  -Class/course for COPD patients with psychosocial problems | -Education of professionals and practice nurses in recognizing psychosocial problems and screening for stress  -Auditing and feedback on quality of life and NHG standard indicators | -Sounding board for COPD patients for discussion and progress in the DMP  -Core team (GP and practice nurse) can be contacted for quality of care  -Training of practice nurses in early recognition of psychosocial problems and referrals to mental health care  -Regular follow-up  -Semi annual screening for distress  -Cooperative agreements  -Regional transmural patient file (EPD) accessible to professionals and patients with additional COPD protocol |
| 17 | Diabetes | Zeist | -Education of patients  -E-health programmes to improve preventive behaviour, healthy lifestyle and self-management skills | -Implementation of NHG care standard for diabetes  -Education of professionals  -Auditing and feedback on patient satisfaction | -Involvement of patient associations  -Delegation of care from GP to practice nurse  -Hospital management of diabetic care, dietary counselling, and eye care  -Consultations with dieticians for newly diagnosed diabetics  -Consultations with diabetic nurses and dieticians for new insulin users  -Regular follow-up  -Multidisciplinary trans-mural cooperation and cooperative agreements between primary and hospital care  -ICT infrastructure to support e-health programme with multi-user functionality and patient portal |
| 18 | Diabetes | Den Haag | -Patient version of the diabetes standard of care  -E-health programmes for patients to keep a logbook and access their files  -Lifestyle programmes  -Exercise programmes | -Educational meetings with other partners in the care chain to transfer knowledge  -Education of GPs in quality of diabetic care  -Education of practice nurses  -Auditing and feedback on process and outcome indicators based on the care standard and benchmarking  -Feedback meetings to discuss results of visitations build relationships among partners | -Special policy for Surinamese and Hindu people  -Multidisciplinary cooperation between primary and hospital care |
| 19 | Diabetes | Nieuwegein* | -Informational meetings  -Informational health fair  -Self-management course: 10–12 patients are trained in medical and psychosocial aspects of self-care  -Minor and intensive support groups | -Implementation of the care standard for diabetes  -Training for practice nurses, diabetes nurses, and dieticians in teaching self-management  -Auditing and feedback on patients at individual and group levels (patients enrolled in the self-management course/support group consultations)  -Auditing and feedback on quality according to patient experiences | -Auditing of patients’ experiences of quality of care  -Redefinition of care process leading to care differentiation in three patient groups: self-managers, patients with minor support, and patients with intensive support  -Regular follow-up (intensity depends on patient group)  -ICT system with patient portal |
| 20 | Depression | Eindhoven | -Motivational interviewing  -Post-diagnostic stepped care: group and individual interventions, internet courses  -E-consultations | -Implementation of NHG standard for depression  -Education of professionals in interventions, motivational interviewing and Beck Depression Inventory (to distinguish between mild and severe depression)  -Auditing and feedback with bottleneck analyses, evaluation of intervention effectiveness, benchmarking, focus group discussions with patients on care evaluation, and evaluative meetings between GPs | -Client board  -Focus group discussion with patients  -Delegation of care from GP to mental health practice nurse  -GP or mental health practice nurse diagnosis of patients and provision of treatment (stepped care) within 2 weeks  -Mental health practice nurse provision of psychological education (part of group intervention)  -Physical therapist provision of exercise programme (part of group intervention)  -Psychologist management of internet course  -GP and/or mental health practice nurse provision of individual interventions  -Email contact with patients  -Stepped care method can be adapted to the demands of each individual practice  -Depression screening among elderly and chronically ill patients  -ICT system accessible to involved professionals  -Internet self-management tool |
| 21 | Psychotic disorders | Den Bosch | -Implementation of family interventions | -Information module on psychotic disorders for relevant partners (schools, police, social workers)  -Training of involved professional on alert plan  -Implementation of multidisciplinary guidelines on schizophrenia  -Auditing and feedback through routine outcome monitoring of treatment outcomes  -Auditing and feedback on patient, programme, and organisational levels | -Development and implementation of alert plans  -Active community treatment team responsibility for intensive treatment, care, and addiction treatment  -Connection with internal and external partners within the mental health chain, improved transition of care from critical to a more stabilized situation |
| 22 | Eating disorders | Leidschendam* | -Preventive public education through websites, guest lecturers at schools; provision of information to GPs  -Educate patients on eating disorders  -Distribution of signal cards to parents  -Self-management enhancement through internet, self-support groups, buddy contact with an experienced professional, regular general assessment, psychiatric home care, and regular outpatient treatment | -Training of professionals in enhancing patients’ self-management  -Auditing and feedback on patients’ weight, eating behaviours, and moods | -Assessment of patient satisfaction to develop further approaches  -Involvement of people with eating disorders in the design and development of a programme; discussion of prevention and self-management  -Regular follow-up  -Early recognition of people with eating disorders |

# Notes: DMP = disease management programme; CVD = cardiovascular disease; EPD = electronic patient dossier; GP = general practitioner; NHG = Dutch GPs association; ICT = information and communications technologies; CBO = Dutch Institute for Quality Improvement; DBC = documentation-based care; COPD = chronic obstructive pulmonary disease; GOLD = Global initiative for chronic obstructive lung disease; *qualitative research case.
